# P05.50. CONSORT extension for N-of-1 trials (CENT) guidelines

**DOI:** 10.1186/1472-6882-12-S1-P410

**Published:** 2012-06-12

**Authors:** L Shamseer, M Sampson, C Bukutu, N Barrowman, D Altman, D Moher, S Vohra

**Affiliations:** 1 5University of Ottawa Evidence-based Practice Center, Ottawa, Canada; 2 Children’s Hospital of Eastern Ontario, Ottawa, Canada; 3 Centre for Statistics in Medicine, Institute of Health Sciences, Oxford, United Kingdom; 4 University of Ottawa Evidence-based Practice Center, Ottawa, Canada; 5 University of Alberta, Edmonton, Canada

## Purpose

N-of-1 trials have been used in medicine to generate treatment information when evidence from randomized controlled trials (RCTs) is not available or applicable. N-of-1 study design maintains the methodological safeguards provided by RCTs (blinding, randomization and controls) yet avoids the disadvantages associated with large trials. A standardized method of reporting of N-of-1 trials, such as the Consolidated Standards of Reporting Trials (CONSORT), would greatly improve the quality and consistency of trial reports in this area. The objective of this study was to develop a CONSORT Extension for N-of-1 Trials (CENT).

## Methods

Checklist items for the CENT guidelines were derived from three systematic reviews on N-of-1 conduct, analysis and meta-analysis. A structured process of obtaining information from a group of experts to refine and finalize the CENT guidelines was carried out. Two stages of questionnaires asked participants to rate the relative importance of suggested checklist items; the second questionnaire was refined based on feedback from the previous one. Participants included those known to have interests in either RCT reporting or N-of-1 methodology. Items included after the Delphi process were debated and finalized during an in-person meeting.

## Results

There were 44 unique respondents between the two rounds of questionnaires. Based on questionnaire results, topics chosen for discussion at the in person meeting included: terminology (what is “N-of-1”?), randomization and blinding, research vs. clinical care, and analysis and meta-analysis. The CENT checklist was refined and adapted to the format of the most recent CONSORT 2010 statement.

## Conclusion

N-of-1 trials may promote an evidence-based approach to therapy so that families, health care providers and policy-makers can make informed choices, and are relevant to both conventional and complementary and alternative medicine. The CONSORT ‘extension’ will facilitate critical appraisal and interpretation of N-of-1 trials by providing authors with guidance on how to improve reporting.

